# How Does F_1_-ATPase Generate Torque?: Analysis From Cryo-Electron Microscopy and Rotational Catalysis of Thermophilic F_1_

**DOI:** 10.3389/fmicb.2022.904084

**Published:** 2022-05-06

**Authors:** Hiroyuki Noji, Hiroshi Ueno

**Affiliations:** Department of Applied Chemistry, Graduate School of Engineering, The University of Tokyo, Tokyo, Japan

**Keywords:** F_1_-ATPase, molecular motor, single-molecule analysis, structure, chemo-mechanical coupling, torque

## Abstract

The F_1_-ATPase is a rotary motor fueled by ATP hydrolysis. Its rotational dynamics have been well characterized using single-molecule rotation assays. While F_1_-ATPases from various species have been studied using rotation assays, the standard model for single-molecule studies has been the F_1_-ATPase from thermophilic *Bacillus* sp. PS3, named TF_1_. Single-molecule studies of TF_1_ have revealed fundamental features of the F_1_-ATPase, such as the principal stoichiometry of chemo-mechanical coupling (hydrolysis of 3 ATP per turn), torque (approximately 40 pN·nm), and work per hydrolysis reaction (80 pN·nm = 48 kJ/mol), which is nearly equivalent to the free energy of ATP hydrolysis. Rotation assays have also revealed that TF_1_ exhibits two stable conformational states during turn: a binding dwell state and a catalytic dwell state. Although many structures of F_1_ have been reported, most of them represent the catalytic dwell state or its related states, and the structure of the binding dwell state remained unknown. A recent cryo-EM study on TF_1_ revealed the structure of the binding dwell state, providing insights into how F_1_ generates torque coupled to ATP hydrolysis. In this review, we discuss the torque generation mechanism of F_1_ based on the structure of the binding dwell state and single-molecule studies.

## Introduction

The F_1_-ATPase is the catalytic core of the F_o_F_1_ ATP synthase. F_o_F_1_ ATP synthase is the ubiquitous rotary motor enzyme, which is found in the membranes of mitochondria, chloroplasts, and bacteria (Hisabori et al., 2013; Junge and Nelson, 2015; Sielaff et al., 2018; Kuhlbrandt, 2019). When isolated from the F_o_ component, which is embedded in the lipid membrane, F_1_ acts as an ATP-driven motor that rotates the central subunit of the rotor against the stator ring by using free energy derived from ATP hydrolysis (Noji et al., 2017). The chemo-mechanical coupling between ATP hydrolysis and rotation of F_1_ is reversible; when the rotation is forcibly reversed, F_1_ catalyzes ATP synthesis reaction (Rondelez et al., 2005). This reversible feature discriminates F_1_ from other molecular motors. However, it should be noted that there are arguments on whether F_1_ synthesizes ATP exactly following the reverse reaction pathway for ATP hydrolysis in the whole F_o_F_1_ complex under ATP synthesis conditions (Vinogradov, 2019). The minimum structure that functions as a rotary motor is the *α*_3_*β*_3_*γ* subcomplex, which is composed of the *α*_3_*β*_3_ stator ring and the rotor *γ* subunit. The *α*_3_*β*_3_ ring possesses three catalytic reaction sites at the α–β interfaces. Most of the catalytic residues are in the *β* subunit, which undergoes large conformational transitions coupled with the catalytic reactions.

The kinetics and physicochemical properties of F_1_ rotation have been well studied in single-molecule rotation assays (Watanabe et al., 2010; Noji et al., 2017). All of the F_1_-ATPases investigated so far show counterclockwise rotation when viewed from the F_o_ side, suggesting that the fundamental mechanism of the chemo-mechanical coupling of F_1_ is highly conserved among species (Konno et al., 2006; Bilyard et al., 2013; Suzuki et al., 2014; Steel et al., 2015; McMillan et al., 2016; Kobayashi et al., 2020; Zarco-Zavala et al., 2020). Since the rotation assay was established (Noji et al., 1997), the *α*_3_*β*_3_*γ* subcomplex of F_1_-ATPase from thermophilic *Bacillus* sp. PS3, which we hereafter call TF_1_ for simplicity, has been intensively studied to reveal fundamental features of the chemo-mechanical coupling reaction of the F_1_-ATPase. Such features include a unitary step size (120°), a coupling stoichiometry of hydrolysis of 3 ATP molecules per turn, a generation of torque against viscous friction (approximately 40 pN·nm), and a high reversibility of the reaction that results in a high efficiency of energy conversion (Yasuda et al., 1998; Rondelez et al., 2005; Hayashi et al., 2010; Toyabe et al., 2011).

**Figure 1:**
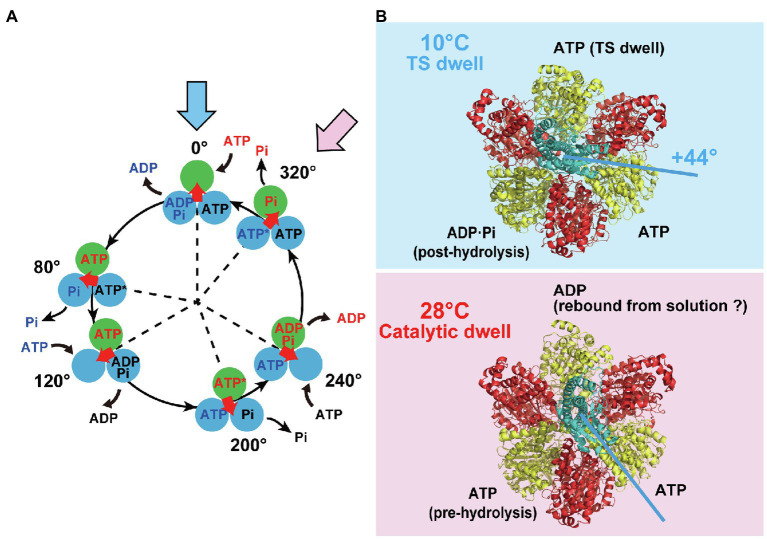
Reaction scheme and cryo-EM structures of TF_1_. **(A)** Reaction scheme of TF_1_. Three circles represent catalytic states of the β subunit, and the arrow at the center indicates the orientation of the *γ* subunit. Blue and pink arrows indicate a binding dwell state and a catalytic dwell state, respectively. **(B)** The cryo-EM structures of TF_1_ obtained at 10°C (highlighted in blue) or at 28°C (highlighted in red). The 10°C and 28°C structures represent the binding dwell state paused by temperature-sensitive reaction and the catalytic dwell state paused by hydrolysis reaction, respectively. The *α*, *β*, and *γ* subunits are represented in red, yellow, and cyan, respectively.

Figure 1A shows our proposed reaction scheme for rotary catalysis by TF_1_ (Watanabe et al., 2010). Each *β* subunit completes a single ATP hydrolysis coupled with a single *γ* rotation. The unitary rotation step is 120° rotation, which is divided into 80° and 40° substeps, initiated after the binding dwell and catalytic dwell, respectively; TF_1_ undergoes three binding dwells and three catalytic dwells in a single 360° rotation of *γ*. Each 80° substep is triggered after ATP binding, and the 40° substep is initiated after hydrolysis of bound ATP. TF_1_ also conducts product-releasing reactions associated with the binding and catalytic dwells; ADP is released at the end of the binding dwell or during the 80° substep, and inorganic phosphate (Pi) is released during the catalytic dwell. The temperature-sensitive (TS) reaction, which is considered to be a conformational rearrangement of the *β* subunit before or after ATP binding, was also found to occur at a binding angle before the 80° substep (Enoki et al., 2009). Thus, TF_1_ conducts multiple reactions in each dwell: ATP binding, ADP release, the TS reaction during the binding dwell, and ATP hydrolysis and Pi release during the catalytic dwell. The rotational position of the *γ* subunit is defined as 0° for one of the *β* subunits in the ATP-waiting state at the binding angle; this is shown in the uppermost panel in the scheme. This *β* subunit then hydrolyzes the bound ATP into ADP and Pi at +200°, releases ADP at +240°, and releases Pi at +320°. The TS reaction is suggested to occur at 0°. Note that the other two *β* subunits follow the same reaction process, although their reaction phases always differ by +120° or + 240°.

TF_1_ has two stable conformational states during catalysis: the binding dwell and the catalytic dwell. Other types of F_1_ have also been reported to have binding and catalytic dwells (Bilyard et al., 2013; Suzuki et al., 2014; Steel et al., 2015; McMillan et al., 2016; Kobayashi et al., 2020). The exception is F_1_ from *Paracoccus denitrificans*, which shows binding and catalytic dwells at the same angles (Zarco-Zavala et al., 2020). It is worth mentioning that mammalian mitochondrial F_1_’s show the third short dwell (Suzuki et al., 2014; Kobayashi et al., 2020), suggesting the additional stable conformational state in mammalian F_1_’s. Since the first crystal structure of F_1_ was published (Abrahams et al., 1994), many different F_1_ structures have been determined under a wide variety of conditions. Most of them, irrespective of species, represent the catalytic dwell state or its related states including the transition states and the inhibited states, in which two of the three *β* subunits assume a closed form (C) with a bound nucleotide (ADP or ATP analog). The helical C-terminal domain of the *β* subunit swings inward in the C conformation, seemingly pushing the *γ* subunit. The third *β* subunit assumes an open form (O), generally without a bound nucleotide, although some structures have shown bound ADP (Menz et al., 2001) or the Pi analog thiophosphate (Bason et al., 2015). This feature is well conserved among the structures of F_1_ in the catalytic dwell state or its related states. As a result, the angular orientation of the *γ* subunit is not significantly different among these structures within the 40° substep (Okazaki and Takada, 2011). Thus, although the F_1_ structures show three conformational states of the *β* subunit in the catalytic dwell (at 80°, 200°, and 320°), the remaining three states of the binding dwell (at 0°, 120°, and 240°) have not been available.

## Cryo-EM Analysis of TF_1_ in the Binding Dwell State

To determine the structure of TF_1_ in the binding dwell state, structural analysis of the mutant TF_1_ (*β*E190D) at a low temperature was conducted using cryogenic electron microscopy (cryo-EM; Sobti et al., 2021). In this analysis, TF_1_ complexes were incubated with MgATP at the low temperature of 10°C to keep the molecules in the binding dwell state while waiting for the TS reaction. A previous single-molecule study revealed that the *β*E190D mutant has an exceptionally long dwell at the binding dwell angle while waiting for the TS reaction to occur (Enoki et al., 2009). For comparison, the structure of the mutant F_1_ at 28°C was also determined, at which temperature the catalytic dwell state is predominant. Cryo-EM analysis of the mutant TF_1_ at 28°C showed a structure corresponding to the catalytic dwell state with the C-C-O conformation of *β*. Although the 28°C structure was almost identical to the catalytic dwell structures of other types of F_1_, one difference was that ADP was bound to *β* in the O conformation, as discussed later. The structure of F_1_ obtained at 10°C showed distinctive conformations of the *β* subunit; two of the *β* subunits adopted intermediate conformations between O and C, while the third one assumed a C conformation. The *β* subunit in the C conformation bound ATP, representing a 120° state according to the reaction scheme (Figure 1A). The *β* subunits in intermediate conformations were half-opened (HO) or half-closed (HC). The HO *β* subunit bound ADP and Pi, thus representing the post-hydrolysis form that should be in the 240° state. The HC *β* subunit had a weakly bound ATP with fewer interacting residues; it should correspond to the 0° state. The resultant arrangements of the 0°, 120°, and 240° states in the F_1_ complex are consistent with the expected arrangements. Moreover, when the 10°C structure was compared to the catalytic dwell structure obtained at 28°C, the *γ* subunit was rotated by +44° from the catalytic dwell state (Figure 1B). This result agrees with the +40° rotation from the catalytic dwell state to the binding dwell state expected from single-molecule studies. Thus, cryo-EM analysis of *β*E190D TF_1_ at 10°C provides the first structural information about F_1_ in the binding dwell state.

## Torque Generation Mechanism

**Figure 2:**
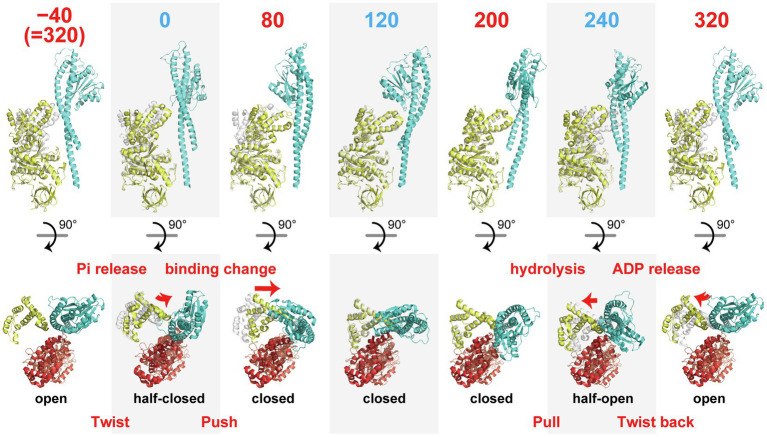
Sequential images of the conformational states of the *β* subunit. αβ-pairs are superimposed on the N-terminal domain (*β*2-82) of the *β* subunit. The upper row shows the conformations of a pair of *β* (yellow) and *γ* subunits (cyan) viewed from the side. The numbers indicate the angle position of the *γ* subunit along the reaction scheme in Figure 1A where 0° is defined as the angle for ATP association. The lower row represents the positions of the C-terminal domains (after P356) of the *β* subunit (yellow) with the *α* (red) and *γ* subunits (blue) viewed from the top. The gray portion shows the structure of the *β* subunit before one reaction state for comparison purposes.

The structure of the binding dwell state enabled us to identify six conformational states of the *β* subunit along the reaction scheme, as shown in Figure 2. Based on this scheme, we now examine the conformational transitions of the *β* subunit revealed by cryo-EM analyses, focusing on a single pair of *β* and *γ* subunits. We then discuss the possible molecular mechanism of torque generation, using all of the available data. Here, the angle represents the angular position of the *γ* subunit during 360° rotation against the *β* subunit in the pair, where the origin, 0°, is defined as the angle at which *β* is in the 0° state, paused at the binding angle.

The 0° state in the HC conformation represents the state after association with ATP and before TS. The transition from −40° (=320°) to 0° induces a change from the O to the HC conformation, which is accompanied by a twisting motion of the C-terminal domain of the *β* subunit. The second large conformational transition is observed in the transition from 0 to 80°, which induces the HC to C transition, which causes a “push” of the C-terminal domain of the *β* subunit toward the off-axis bulge of the *γ* subunit. The transitions from 80 to 120° and 120 to 200° do not involve obvious conformational changes in the *β* subunit. The third large conformational transition is during the C to HO transition from 200 to 240°. This is the “pull back” motion of the C-terminal domain of the *β* subunit from the axis of the *γ* subunit. The final conformational transition is during the HO to O transition from 240 to 320°. This involves a “twist back” motion of the C-terminal domain of the *β* subunit. Thus, the series of conformational transitions of the *β* subunit in the course of a single turnover of ATP hydrolysis can be explained as a “twist” (−40–0°), a “push” (0–80°), a “pull back” (200–240°), and a “twist back” (240–320°) motions.

The first conformational transition, the “twist” motion, is seemingly driven by binding of ATP. Because the binding dwell structure before ATP association is not yet known, it is not clear whether the twist motion is induced by association with ATP at 0° or by the 40° substep rotation before ATP association. In the former case, the *β* subunit conducts the twist motion without driving the rotation of *γ*, and the twist motion is not directly involved in torque generation. In the latter case, the direct cause of the twist motion is not ATP binding but rather another reaction, such as Pi release or a conformational rearrangement not coupled with a particular catalytic reaction. A single-molecule study by Masaike et al. (2008) supports the former model. They observed the rotation of a fluorophore attached to a helix of the C-terminal domain of the *β* subunit and found no obvious difference between the 320 and 0° states (Masaike et al., 2008). Thus, it is likely that the binding dwell structure before ATP association corresponds to the O conformation and the twist motion is triggered by ATP association but not directly coupled with *γ* rotation.

**Figure 3:**
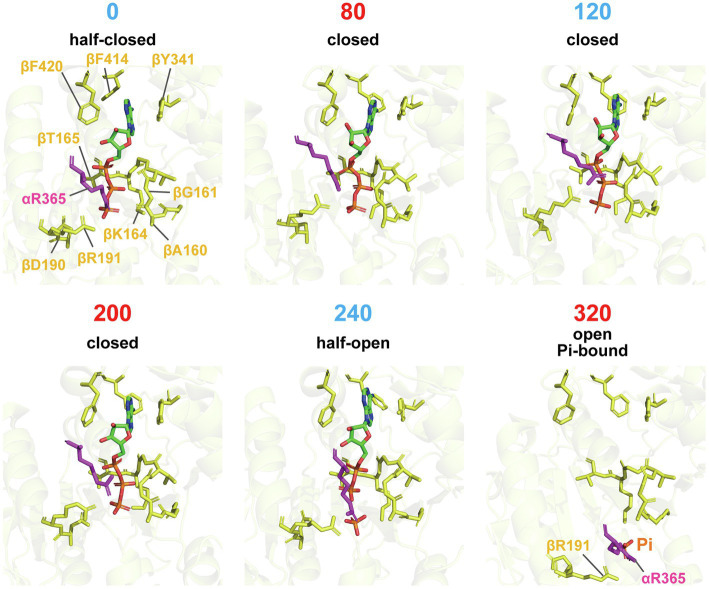
Close-up structures of catalytic sites on the *β* subunit. Residues around the nucleotides of each conformational state from 0° to 320°, superimposed on residues (*β*163-165, *β*190, *β*191, *β*341, *β*414, and *β*420), are shown in stick representation. αR365 is shown in magenta. Note that the Pi-bound structure is used for the 320° conformation.

The “push” motion that occurs from 0 to 80° should be responsible for torque generation. In this conformational transition, the *β* subunit swings its C-terminal domain toward the axis of the *γ* subunit, enveloping the bound ATP. Figure 3 shows a close-up view of the structure around the catalytic site. In comparison with the structure of the 0° state, the structure of the 80° state shows tighter associations of the bound ATP with the surrounding catalytic residues. In particular, Arg365 of the *α* subunit (*α*R365) and R191 of the *β* subunit (*β*R191) shift their position toward the phosphate group of ATP in the 80° state. In addition, the p-loop around *β*G161 approaches the phosphate group of ATP in the 80° state. These features are consistent with the findings of a single-molecule manipulation study that showed that TF_1_ exponentially increased affinity to ATP by 235-fold during rotation by 60° (Watanabe et al., 2011). The torque generated by this affinity change is estimated at 21–54 pN·nm, indicating that the affinity change is the major torque-generating event in the 80° substep. This mechanism of torque generation upon the affinity change is also consistent with the binding-change mechanism proposed by Boyer, who suggested that the proton motive force (PMF) is used mainly to loosen the affinity of the catalytic site for ATP during ATP synthesis (Boyer, 2000). Thus, tightening the affinity for ATP is the major torque-generating step in the rotation of F_1_ driven by ATP hydrolysis.

The third conformational transition, the C-HO transition from 200 to 240°, is associated with hydrolysis of the bound ATP, as the bound nucleotide switches from ATP in the 200° state to ADP and Pi in the 240° state. Many studies have revealed that ATP is hydrolyzed in the 200° state. Because the *β*E190D mutant is significantly retarded in the rate of ATP hydrolysis (Shimabukuro et al., 2003), it is very reasonable to see ATP bound in the 200° state. Thus, 240° represents the post-hydrolysis state. Figure 3 shows a clear difference in the residues associating with Pi, such that *α*Arg365 and *β*R191 undergo a positional shift to separate from the phosphate group of ADP. Although not shown in the figure, the α–β interface opens slightly during the C-HO transition. The aforementioned single-molecule manipulation study also demonstrated that the equilibrium constant of the hydrolysis state (ADP + Pi) against the synthesis state (ATP) increases with rotation (Watanabe et al., 2011), indicating the contribution of hydrolysis for torque generation. However, the estimated torque (4–17 pN·nm) is significantly lower than that made possible by the change in affinity for ATP (Watanabe et al., 2011; Noji et al., 2017).

The molecular mechanism for the fourth conformational transition, the HO-O transition from 240 to 320°, must also be considered. The reaction scheme shown in Figure 1A, which suggests that ADP is released during this transition, is based on other structural analyses that show no bound nucleotides in the 320° state (Abrahams et al., 1994; Bowler et al., 2007) and simultaneous observations of rotation and the association/dissociation of fluorescently labeled nucleotides (Nishizaka et al., 2004; Adachi et al., 2007). However, ADP was bound in the 320° state in the catalytic dwell structure of TF_1_. Considering that this structure was obtained in the presence of 10 mM ATP at 28°C, it is likely that ADP rebinds to the *β* subunit in the 320° state. Figure 3 shows the 320° conformation of the Pi-bound structure of TF_1_ obtained in medium containing 100 mM Pi and no nucleotides (Sobti et al., 2021). The exact determination of the catalytic state at 320° requires further investigation.

The 320 to 360° rotation corresponds to the −40 to 0° rotation. As mentioned above, it is likely that the twist motion during the O-HC transition is not directly coupled with *γ* rotation. Several studies have indicated that the 320 to 360° rotation is coupled with Pi release from the 320° state. The cryo-EM structure of TF_1_ in the catalytic dwell state in the presence of Pi supports this model, showing Pi bound in the 320° state, as seen in the crystal structures of bovine or yeast mitochondrial F_1_ (Kabaleeswaran et al., 2006; Bason et al., 2015). Kinetic analysis of rotation in the presence of Pi suggests that Pi release is the second major torque-generating step in F_1_ in addition to the change in affinity for ATP (Adachi et al., 2007). A comprehensive analysis of the crystal structure suggests that Pi release is coupled with opening of the α–β interface (Okazaki and Takada, 2011). Further structural analysis will be required to elucidate the torque generation mechanism upon Pi release. In this regard, it is worth noting that the cryo-EM structure of TF_1_ in the 320° state with bound Pi reveals a putative Pi exit channel in the *β* subunit. This model awaits experimental and theoretical verification.

## Remaining Issues

The cryo-EM structures of TF_1_ provide several important insights into the molecular mechanism by which F_1_ couples a catalytic reaction to torque generation. However, cryo-EM study on the mutant TF_1_ also highlights several important issues that must be addressed. One is the molecular mechanism of the conformational entrapment of the *β* subunit in HC state that leads TS dwell. The current structural information in addition to previous single-molecule studies are not sufficient to deduce this mechanism. Molecular dynamics simulation of TF_1_ starting from TS dwell could address this point. Another issue is the structure of the 0° state before ATP association. The structure, once known, will resolve whether the *β* subunit assumes a conformation different from the HC state before ATP association and also show what type of conformational change is coupled with Pi release. Further structural analysis of TF_1_ in its catalytic dwell state will be required to determine the point at which ADP is released. The cryo-EM study also provides a new way to address the structural basis of F_1_ allostery. TF_1_ is sufficiently stable to maintain the *α*_3_*β*_3_ ring structure even when the *γ* subunit is removed. A previous study using high-speed atomic force microscopy showed that the isolated *α*_3_*β*_3_ ring still possesses allostery (Uchihashi et al., 2011); the *β* subunits in the *α*_3_*β*_3_ ring show sequential power-stroking motions, demonstrating that the fundamental allostery of F_1_ originates from the *α*_3_*β*_3_ structure. Currently, only the nucleotide-free structure of *α*_3_*β*_3_ has been reported, in which all of the *β* subunits are in the O state. A cryo-EM approach would allow structural analysis of the *α*_3_*β*_3_ ring with bound nucleotides, which should break the structural symmetry of the ring and provide important insights into the basis of the fundamental allostery of the *α*_3_*β*_3_ ring. A more fundamental question arising from a comprehensive point of view is the generality of the findings; how well the molecular mechanism of torque generation learned from the studies on TF_1_ is conserved among other F_1_’s. Considering the many common features found in the structure and rotation dynamics, it seems that the fundamental mechanism should be conserved across species. However, some points would be diverse to meet physiological requirements. In this context, it would be very intriguing to analyze how the torque generation mechanism is conserved or diverse between F_1_- and V_1_-ATPase. The manner of the conformational transition as well as the torque generation mechanism of binding-change process of V_1_-ATPase were reported to be different from those of F_1_, and even different among V_1_-ATPase’s (Imamura et al., 2005; Arai et al., 2013; Tirtom et al., 2013; Iida et al., 2019; Singharoy et al., 2019; Kishikawa et al., 2022). Therefore, more comprehensive investigation on the torque generation mechanism of V_1_ is required.

## Author Contributions

HN and HU contributed to the conceptualization, review, and editing. HN wrote the original draft of the manuscript. All authors have contributed to the manuscript and approved the submitted version.

## Funding

This work was supported in part by a Grant-in-Aid for Scientific Research on Innovation Areas (JP19H05380 and JP21H00388 to HU), Grant-in-Aids for Scientific Research (S; JP19H05624 to HN) from the Japan Society for the Promotion of Science, and JST CREST, Japan (JPMJCR19S4 to HN).

## Conflict of Interest

The authors declare that the research was conducted in the absence of any commercial or financial relationships that could be construed as potential conflicts of interest.

## Publisher’s Note

All claims expressed in this article are solely those of the authors and do not necessarily represent those of their affiliated organizations, or those of the publisher, the editors and the reviewers. Any product that may be evaluated in this article, or claim that may be made by its manufacturer, is not guaranteed or endorsed by the publisher.
